# Assessment of quadriceps muscle mass by ultrasound in the postoperative period of cardiac surgery

**DOI:** 10.1186/s13089-023-00348-z

**Published:** 2024-02-12

**Authors:** Nestor David Caicedo Buitrago, Diana Trejos Gallego, Maria Cristina Florián Pérez, Carlos Andrés Quintero Cardona, Cristian Chaparro Botero

**Affiliations:** 1 Unidad de Cuidados Intensivos, E.S.E. Hospital Departamental Universitario de Caldas Santa Sofía, Manizales, Colombia; 2 Unidad de Cuidados Intensivos, Clínica San Marcel, Manizales, Colombia; 3 Facultad de Ciencias de la Salud, Especialización en Medicina Crítica y Cuidado Intensivo, Universidad de Manizales, Manizales, Colombia; 4 Unidad de Cuidados Intensivos S.E.S. Hospital Universitario de Caldas, Manizales, Colombia

**Keywords:** Intensive care, Ultrasound, Cardiac surgery, Skeletal muscle, Quadriceps

## Abstract

**Background:**

Patients undergoing cardiac surgery are exposed to many factors that activate catabolic and inflammatory pathways, which affect skeletal muscle and are, therefore, related to unfavorable hospital outcomes. Given the limited information on the behavior of muscle mass in critically ill patients, the objective of this study was to evaluate the impact on quantitative and qualitative measurements of quadriceps muscle mass using ultrasound after cardiac surgery. To accomplish this, a prospective, descriptive, and correlational study was conducted at a tertiary care hospital. Quadriceps muscle mass was evaluated via ultrasound in 31 adult patients in the postoperative period of cardiac surgery, with daily follow-up until postoperative day 7, as well as an assessment of associations with negative outcomes at 28 days.

**Results:**

A 16% reduction in the cross-sectional area of the rectus femoris was found (95% CI 4.2–3.5 cm^2^; *p* 0.002), as well as a 24% reduction in the pennation angle of the rectus femoris (95% CI 11.1–8.4 degrees; *p*: 0.025). However, changes in the thickness of the rectus femoris, vastus internus, vastus lateralis, the length of the fascicle of the vastus lateralis, the pennation angle of the vastus lateralis, the sarcopenia index, and the Hekmat score were not statistically significant. There was no significant association between quadriceps muscle mass measurements and Intensive Care Unit (ICU) length stay or 28-day mortality.

**Conclusions:**

Patients in the postoperative period of cardiac surgery evaluated by ultrasound exhibit both quantitative and qualitative changes in quadriceps muscle mass. A significant reduction in muscle mass is observed but this is not associated with unfavorable outcomes.

## Background

Cardiovascular disease is the leading cause of death worldwide, with an estimated 32% of those affected potentially requiring cardiac surgical intervention [1]. Cardiac surgical procedures are considered high-risk surgeries due to their relationship with multiorgan failure. Therefore, postoperative care involves vigilant and comprehensive management in an Intensive Care Unit (ICU). In addition, there is extensive literature showing a decline in health-related quality of life during the postoperative period, which in turn has an impact on mortality [2].

It is common to observe a reduction in the functional capacity of patients after their admission to an ICU. Factors such as immobility, inflammation, mechanical ventilation, and sedation can contribute to the development of acquired neuromuscular weakness in the ICU, a condition observed in up to 25% of critical illness survivors [3]. Muscle mass loss is a significant factor in this process, as it is associated with decreased functionality and quality of life [4], further leading to prolonged ICU stays and increased mortality [5]. After cardiac surgery, weight loss can be observed in up to 95% of patients, concomitant with a sustained inflammatory response, depression, and reduced appetite, which impact physical health and functional status [6].

Estimation of body composition, specifically muscle mass, in critically ill patients is a challenge. Currently, there is no reliable tool available to quantify and monitor muscle tissue in these patients. Various methods have been tested for the quantification of muscle mass in critical patients, such as bioelectrical impedance analysis (BIA) [7], computed tomography (CT) [8], dual-energy X-ray absorptiometry (DEXA) [9], nuclear magnetic resonance (NMR) [10], and ultrasound. Among these methods, ultrasound has been proposed as a promising tool with ongoing trials that seem to promise better evidence [11].

As far as it is known, there is scarce evidence regarding the behavior of muscle mass during the perioperative period of cardiac surgery. Therefore, we aim to possibly establish a landmark to the impact of the surgical procedure on the quadriceps muscle mass using ultrasound.

## Methods

This is a prospective, descriptive, and correlational study conducted at a tertiary center in the city of Manizales, Caldas Department, Colombia. After obtaining approval from the ethics committee of the Hospital Departamental Santa Sofía, patients over 18 years of age with an indication for cardiac surgery who were admitted to the ICU postoperatively were identified. Such patients underwent invasive blood pressure monitoring, strict multiparametric clinical surveillance, invasive mechanical ventilation, vasopressors, and inotropes, and in specific cases, advanced hemodynamic monitoring.

During the preoperative period, data on each patient's age, gender, and relevant medical history were collected. Anthropometric measurements such as height, weight, and body mass index (BMI) were estimated. In addition, risk assessment scales were applied, including EuroScore II, Barthel, Charlson, Global Leadership Initiative on Malnutrition (GLIM) criteria, and the Clinical Frailty Scale (CFS). This scale allows the patient to be assigned to 1 of 9 categories considering activity, motivation, dependence, and chronic disease control. In this way, it classifies patients into nine categories, ranging from very fit (CFS = 1) to terminally sick (CFS = 9) [12]. Muscle strength was evaluated using the Medical Research Council (MRC) scale.

On the first postoperative day, data related to the surgical procedure (type of surgery, duration of surgery, duration of extracorporeal circulation) were recorded. Relevant management information, such as the need for vasoactive drugs, analgesia, sedation, ventilatory support, nutritional support, glucose measurements, insulin requirements and active delirium screening using the Confusion Assessment Method (CAM–ICU) scale, were also documented.

Muscle strength was monitored using the MRC scale based on the patients' collaboration status. Subsequently, ultrasound examination of the quadriceps muscle was performed. These variables were assessed every other day (days 1, 3, 5, 7) until the seventh day of ICU stay or until discharge. In addition, outcomes at 28 days, mortality, ICU length of stay, and duration of invasive mechanical ventilation were recorded.

### Ultrasound protocol

The ultrasound assessments were conducted by a critical care specialist (ultrasound senior training) and two critical care residents (ultrasound junior training) with more than 5 and 2 years of experience in ultrasound, respectively. A high-frequency linear transducer in musculoskeletal mode (Philips Ultrasound, Bothell, WA) with factory harmonics settings was used. Ultrasound gain was set between 50 and 70%, and the depth was adjusted to visualize the femoral cortex or the deep aponeurosis of the muscle group under evaluation, using mode B to obtain measurements.

To assess the rectus femoris, the distance between the right anterosuperior iliac spine and the right superior patellar border was measured using a rigid tape measure. Marks were made at the midpoint (50% of the total distance) and the distal third (66.6% of the total distance). To evaluate the vastus lateralis, the medial and lateral borders were delineated by ultrasound in the distal third, and the transverse measurement was made with a tape measure, marking the midpoint on the skin.

Three quantitative measurements were recorded: (1) the rectus femoris cross-sectional area (RFCSA) in cm^2^, (2) the rectus femoris and vastus internus thickness (RFVIT) in cm, and (3) the vastus lateralis thickness (VLT) in cm. In addition, three qualitative measurements were performed: (1) the pennation angle of the vastus lateralis (VLPA) in degrees, (2) the length of the fascicle of the vastus lateralis (VLFL) in cm, and (3) the Hekmat Score (HS).

The evaluation of RFCSA followed the proposal by Puthucheary et al. [11], the quadriceps thickness (QT) was obtained following the protocol of the VALIDUM study [13], the measurement of the pennation angle of the rectus femoris (RFPA) was based on the work by Ryochi et al. [14], the VLT, VPLA, and estimation of the sarcopenia index followed the recommendations of Narici et al. [15], and the HS was scored according to the analysis by Grimm et al. [16].

### Quantitative and qualitative measurements of quadriceps muscle mass

The RFCSA was measured in the distal third of the right lower limb using a transducer in the transverse axis and abundant transduction gel. A view of the muscle with minimal compression was obtained, and the image was frozen. The area in cm^2^ was delimited using the planimetry function of the ultrasound machine's software. Three consecutive measurements were taken, and the average was recorded [11].

The RFVIT was measured in the mid and distal third in the transverse axis with maximum compression. The image was frozen, and the distance between the femoral cortex and the lower boundary of the superior fascia of the rectus femoris was identified. Each point was scanned twice, and the average measurement was recorded [13].

The RFPA was measured in the distal third. It was scanned in a longitudinal axis with minimal compression and placed parallel to the skin. Then, it was tilted laterally between 15° and 30°. The image was frozen, and the angle of the fascicles with the inferior aponeurosis was calculated using the equipment's angle tool [14].

**Fig. 1:**
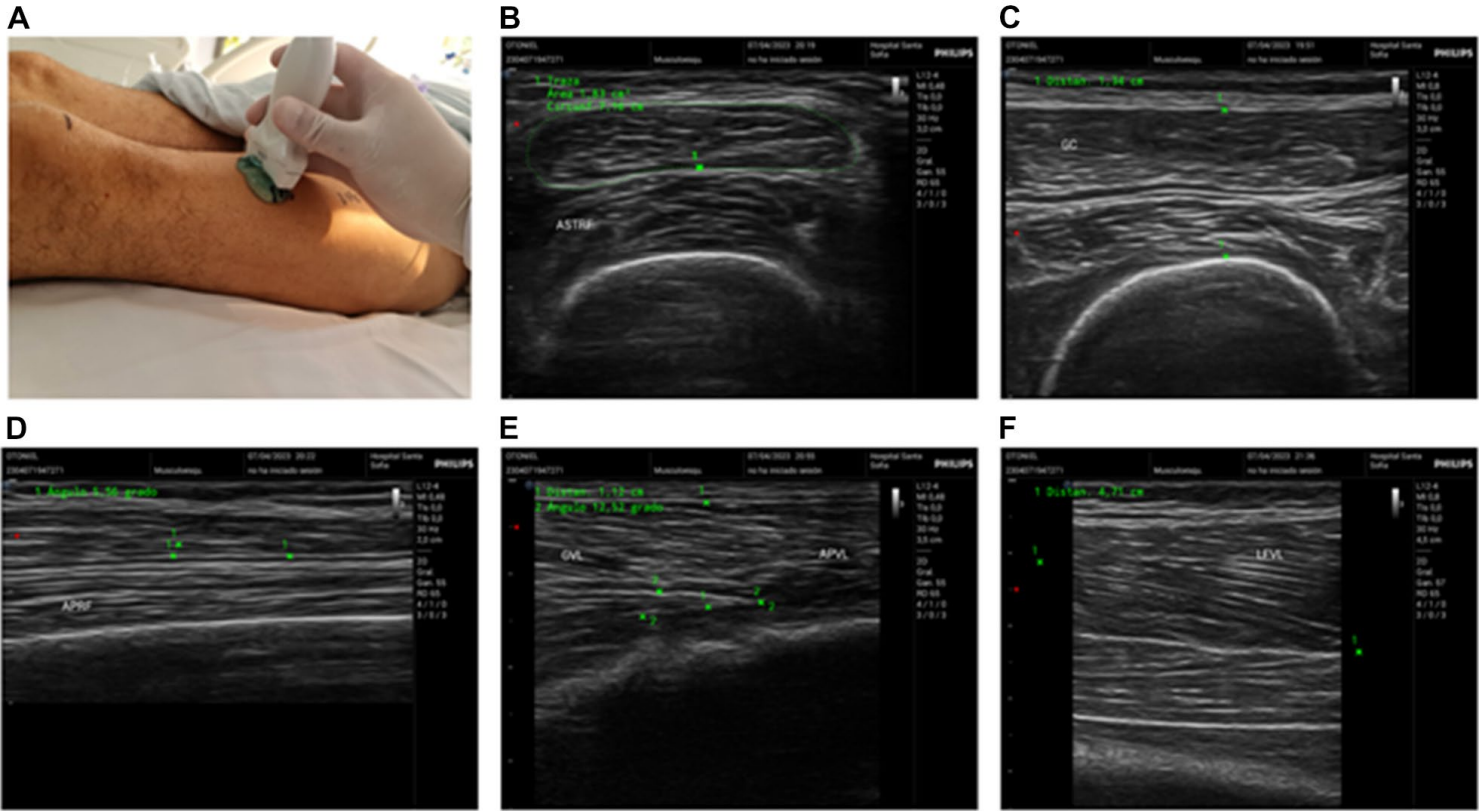
Assessment of quantitative and qualitative measurements of the quadriceps using ultrasound. **A** Measurement of the midpoint and distal third of the quadriceps, skin marking for sequential measurements, and muscle evaluation in the transverse axis with a high-frequency linear transducer. **B** Cross-sectional area of the rectus femoris. **C** Thickness of the rectus femoris and vastus internus. **D** Pennation angle of the rectus femoris. **E** Thickness and pennation angle of the vastus lateralis. **F** Length of the fascicle of the vastus lateralis using a linear extrapolation technique

The VLT and VLFL were also measured in the distal third with the transducer in the transverse axis and minimal compression. The borders of the rectus femoris were delineated, and the midpoint was selected. The transducer was directed laterally to improve the visualization of the central area of the vastus lateralis, and the orientation was changed to the longitudinal axis. The thickness and length of the fascicle (FL) were measured, and the pennation angle was also measured. The sarcopenia index was calculated using the formula FL/thickness [15]. Figure 1 shows the obtained measurements.

### Statistical analysis

Quantitative variables were described using mean, median, standard deviation (SD), minimum, and maximum, while qualitative variables were presented as frequency and percentage. The Shapiro–Wilk statistic value is presented, and for those variables that exhibit a normal distribution, the 95% confidence interval for the mean is described. If such measurement was not possible, the same interval is provided for the median.

Correlation analysis was carried out for quantitative variables, categorized by time, using Pearson's coefficient if the variables followed a normal distribution, or Spearman's coefficient if not. Changes over time for each quantitative variable were established using repeated-measures ANOVA. Logistic regression was performed for each timepoint to establish models explaining mortality based on different quantitative and qualitative variables related to quadriceps muscle mass.

Statistical inference was conducted at a significance level of 5% using the Jamovi version 2.2 statistical software. To determine effect size and test power, the previously mentioned statistical package and Gpower version 3.1.9.6 were employed. The sample for this study was selected using a convenience sampling method, based on the inclusion of individuals who underwent cardiac surgery at Santa Sofia Hospital during the months of August and September 2022. Therefore, the analysis of effect size and power was conducted post hoc.

## Results

From August 1st to September 30th, 2022, data from 31 adult patients who underwent cardiac surgery were collected. Among them, 10 were female (32.3%) and 21 were male (67.7%). The mean age of the group was 61.9 ± 12.5 years, with no statistically significant difference between males and females (*p* = 0.916). The youngest patient was 29 years, while the oldest was 77 years, indicating sample homogeneity, except for EuroScore II (Table 1).

**Table 1:** Descriptive statistics of the variables evaluated in the preoperative period

Statistic	Sex	Age (years)	Weight (kg)	Height (cm)	BMI (kg/m^2^)	Euroscore II Scale
N	W	10	10	10	10	10
	M	21	21	21	21	20
Mean	W	62.6	65.7	160	25.5	2
	M	61.6	70.9	167	25.2	2.38
CI 95% LL	W	54.4	55.5	157	21.8	1.11
	M	–	65.2	164	23.7	–
CI 95% UL	W	70.8	75.9	163	29.1	2.9
	M	–	76.6	170	26.8	–
SD	W	11.5	14.3	4,5	5.08	1.25
Median	M	13.2	12.5	7.12	3.35	1.73
CI 95% lower limit	W	65.0	66.5	160.0	25.4	1.79
	M	65.0	68.0	165.0	25.0	1.97
CI 95% upper limit	W	–	–	–	–	–
	M	61.0				1.51
CV (%)	W	–	–	–	–	–
Shapiro–Wilk	M	67.0	–	–	–	2.39

W, Women; M, Man; CI95%LL, Confidence Interval 95% Lower Limit; CI95%UL, Confidence Interval 95% Upper Limit; SD, Standard Deviation; BMI, Body Mass Index; CV, Coefficient of Variation

Barthel Index was 100 for 96.8% of the patients, while the remaining 3.2% had a score of 90. Charlson Index indicated that 32.3% of the patients had values of three or higher on this scale. The Fragility Index classified 29 individuals in level II (93.5%) and two in level I (6.5%). The most common medical history included hypertension in 70.9% of patients and diabetes mellitus in 16.1%.

Regarding the GLIM criteria upon admission, the majority of patients did not meet malnutrition criteria (87.1%). Coronary artery disease and mitral insufficiency were the main reasons for surgical interventions, accounting for 51.6% and 22.6%, respectively. As a result, most procedures involved coronary artery bypass grafting and mitral valve replacement.

On the day of surgery, the mean duration of extracorporeal circulation was 78.5 ± 24.6 min, and the mean aortic cross-clamp time was 72.1 ± 19.5 min. On average, patients stayed in the ICU for 8.9 ± 6.3 days, with a minimum of 3 days and a maximum of 29 days, with 5 days being the most frequent value. In addition, the mean duration of invasive mechanical ventilation was 2.4 ± 3.5 days, and only 18 patients required ventilation for 1 day.

During the analyzed days, the use of vasoactive drugs was generally low, with 83.9% and 80.6% of patients being weaned off vasopressor and inotropic support, respectively, by day 3. Regarding respiratory support, 79% of patients were successfully extubated within the first 24 h. CAM–ICU scale was positive for delirium in up to 12.9% of patients on day 7. For nutritional support, 80.6% of patients were tolerating oral intake well by day 3. In terms of muscle strength, assessed using the MRC scale, 80.6% of patients had a score of 60 on day 3, which decreased to 13% having a score lower than 54 on day 7.

**Fig. 2:**
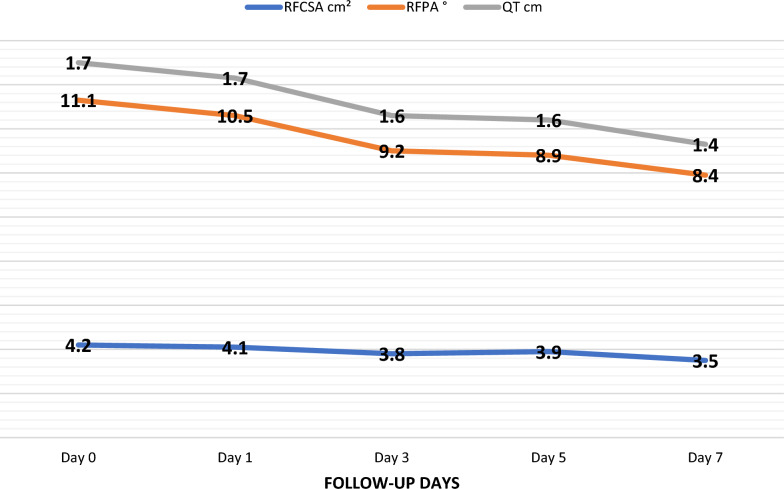
Muscular mass behavior in the interdaily follow-up. RFCSA, Rectus Femoris Cross-Sectional Area; RFPA, Rectus Femoris Pennation Angle; QT, Quadriceps Thickness

Regarding the ultrasound evaluation of the quadriceps, QT, VLT, RFCSA, and RFPA showed a decreasing trend each day (Fig. 2), while VLFL and VLPA tended to increase with the passage of days (Table 2). The HS remained relatively stable, and the sarcopenia index showed an unstable behavior.

**Table 2:** Statistics for the evaluated variables on days 0, 1, 3, 5, and 7

Variable	Days	N	Min	Max	Mean	SD	95% LL	95% UL	Med
Rectus femoris cross-sectional area (cm^2^)	0	31	2.2	7.1	4.2	1.3	3.8	4.7	4.2
	1	31	2.2	7.2	4.1	1.3	3.6	4.5	4.1
	3	31	2.0	7.2	3.8	1.1	3.4	4.2	3.6
	5	19	1.8	7.2	3.9	1.5	3.2	4.5	3.6
	7	10	2.1	5.3	3.5	1.1	2.8	4.2	3.4
Rectus femoris pennation angle (degrees)	0	31	5.4	19.9	11.1	3.9	9.8	12.5	11.4
	1	31	6.2	16.1	10.5	3.0	9.5	11.6	10.2
	3	31	2.7	13.0	9.2	2.3	8.4	10.0	9.8
	5	19	2.4	15.4	8.9	3.2	7.5	10.3	8.6
	7	10	2.1	14.0	8.4	3.4	6.3	10.5	7.8
Rectus femoris thickness (cm)	0	31	1.1	4.2	1.7	0.6	1.5	1.9	1.5
	1	31	1.0	3.2	1.7	0.5	1.5	1.9	1.5
	3	31	1.1	3.1	1.6	0.5	1.4	1.8	1.5
	5	19	1.0	2.8	1.6	0.5	1.4	1.8	1.4
	7	10	1.1	2.1	1.4	0.3	1.2	1.6	1.4
Vastus lateralis thickness (cm)	0	31	1.0	3.2	1.8	0.5	1.6	2.0	1.8
	1	31	1.1	3.1	1.9	0.5	1.7	2.0	1.9
	3	31	1.0	2.4	1.7	0.4	1.6	1.9	1.7
	5	19	0.9	2.4	1.6	0.5	1.4	1.8	1.7
	7	10	1.0	2.2	1.5	0.4	1.2	1.8	1.4
Vastus lateralis fascicle length (mm)	0	31	4.0	9.8	6.6	1.4	6.1	7.1	6.4
	1	31	43	9.8	6.7	1.5	6.2	7.2	6.4
	3	30	4.8	9.7	6.8	1.4	6.3	7.3	6.3
	5	19	4.1	12.0	6.7	1.9	5.8	7.5	6.6
	7	10	4.2	9.5	6.9	1.7	5.8	8.0	7.2
Vastus lateralis pennation angle (degrees)	0	31	6.9	26.0	15.7	4.1	14.3	17.1	15.0
	1	31	7.0	25.0	15.9	4.7	14.2	17.5	16.6
	3	31	7.5	23.0	14.4	4.0	13.0	15.8	14.1
	5	19	5.9	25.0	13.1	4.3	11.2	15.0	12.3
	7	10	10.0	20.0	14.6	3.2	12.6	16.6	14.0
Sarcopenia index	0	31	2.5	6.6	3.9	1.1	3.5	4.3	3.7
	1	31	2.4	4.8	3.7	0.6	3.4	3.9	3.6
	3	30	2.5	6.6	4.1	1.0	3.8	4.5	4.0
	5	19	2.5	6.3	4.3	1.0	3.8	4.7	4.5
	7	10	0.7	6.6	4.2	1.6	3.2	5.1	4.1

Min, Minimum; Max, Maximum; SD, Standard Deviation; 95% CI LL, 95% Confidence Interval Lower Limit; 95% CI UL, 95% Confidence Interval Upper Limit; Med, Median

Through repeated-measures ANOVA, statistically significant differences were found only in the variables RFCSA and RFPA when comparing the preoperative period and the follow-up days. The percentage of patients who experienced a decrease in RFCSA between the preoperative period and day 7 was 90%, and for RFPA, it was 80.6% when comparing day 1 and day 3. The effect size and power analysis revealed that for both RFCSA and RFPA, a power greater than 95% was achieved, whereas for the other variables, except for the sarcopenia index, the effect size was small (Table 3).

**Table 3:** Repeated-measures ANOVA over time

Variable	ANOVA *p* value	Partial eta squared	Power	Compared times		Tukey's *p* value
Rectus femoris cross-sectional area	0.002*	0.364	0.999	d_0_ vs	d_1_	0.091
					d_3_	0.049*
					d_5_	0.013*
					d_7_	0.010*
				d_1_ vs	d_3_	0.201
					d_5_	0.138
					d_7_	0.016*
				d_3_ vs	d_5_	0.356
					d_7_	0.144
				d_5_ vs	d_7_	0.781
Rectus femoris pennation angle	0.025*	0.260	0.965	d_0_ vs	d_1_	0.845
					d_3_	0.209
					d_5_	0.015*
					d_7_	0.130
				d_1_ vs	d_3_	0.285
					d_5_	0.025*
					d_7_	0.138
				d_3_ vs	d_5_	0.044*
					d_7_	0.179
				d_5_ vs	d_7_	0.455
Rectus femoris thickness	0.408	0.092	0.209	–	–	–
Vastus lateralis thickness	0.811	0.061	0.1122	–	–	–
Vastus lateralis fascicle length	0.811	0.047	0.085	–	–	–
Vastus lateralis pennation angle	0.491	0.088	0.190	–	–	–
Index of sarcopenia	0.138	0.190	0.750	–	–	–
Average blood glucose	0.440	0.094	0.200	–	–	–

^*^p value < 0.05. d_0,_ day 0; d_1_, day 1; d_3_, day 3; d_5_, day 5; d_7_, day 7

When logistic regression models were sought to explain the probability of death based on quantitative and qualitative variables at each timepoint, no statistical significance was found for any of them. In other words, the probability of death or prolonged stay in the ICU cannot be explained based on the skeletal muscle measurements obtained through ultrasound.

## Discussion

In this prospective observational study, we evaluated quadriceps muscle mass in patients from a tertiary care center who underwent cardiac surgery, with follow-up using ultrasound examination during the first postoperative week. We observed a progressive reduction in the RFCSA and RFPA with an average loss of 16% and 24%, respectively. These findings indicate a quantitative and qualitative deterioration in skeletal muscle, but they were not associated with ICU stay or increased 28-day mortality.

The European Society for Clinical Nutrition and Metabolism (ESPEN) guidelines for ICU recommend a general physical examination to assess the body composition of critically ill patients and, if possible, estimate muscle mass and strength as a point of good clinical practice [17]. Reduced muscle mass has recently been proposed as one of the phenotypic criteria for the diagnosis of malnutrition [18], but its quantification is infrequent due to the need for trained professionals and specialized body composition methods [19].

Muscle mass evaluation can be approached technically and clinically. The technical approach includes methods, such as Air Displacement Plethysmography (ADP), DEXA, CT, and ultrasound, with difficulties in access to technology and training in interpretation. The clinical approach is based on anthropometry and physical examination, with limitations in validation, cutoff points, and training [20]. Among the technical tools, ultrasound has the highest availability, and muscle mass evaluation can be performed at the patient's bedside without the risks of ionizing radiation.

Patients who develop critical illness often experience functional disability associated with acquired weakness in the ICU, and secondary myopathy involves a heterogeneous pathophysiology, including components of muscle atrophy, altered contractile capacity, proteolysis, reduced mitochondrial content, and impaired regenerative capacity [21]. In patients undergoing cardiac surgery, there is evidence of imbalances in insulin-like growth factor 1 and myostatin concentrations, molecules related to muscle atrophy and hypertrophy [22]. In addition, there is evidence linking preoperative catabolic accelerators with postoperative hypercatabolic state and secondary proteolysis. The association between clinical predictors such as hemoglobin, body mass index, duration of extracorporeal circulation (ECC), and postoperative interleukin-6 concentrations has been investigated in relation to the greater surgical procedure-induced muscle proteolysis [23].

A recent systematic review and meta-analysis by Fazzini et al. [24], evaluated muscle mass loss in the ICU using ultrasound in 85% of the studies and CT in the remaining 15%. This review included 52 studies with 3251 patients and found an approximate 2% loss of skeletal muscle per day during the first week of ICU stay. Other studies have demonstrated up to a 20% loss of muscle mass in the first week of ICU stay using ultrasound [25]. These findings are similar to those in the current study, which showed a reduction in quadriceps muscle mass of up to 24% based on RFPA measurements. However, to the best of our knowledge, there is limited research that has evaluated skeletal muscle by ultrasound in the postoperative period of cardiac surgery.

Among the reported studies is the one conducted by Dimopoulus et al. [26], in which the authors recruited postoperative cardiac surgery patients and measured the global rectus femoris thickness index by ultrasound from postoperative day 1, with follow-up every 48 h until day 6 or until discharge.

They documented a reduction of up to 3.5% of the QT during the evaluation period, and patients with below-average thickness values on day 1 had a longer ICU stay and more days of mechanical ventilation. These results differ from those found in this study, as no association was found between the evaluated muscle mass parameters and ICU stay. However, the QT change in the current study agrees with the one reported by Dimopoulus, as the percentage of patients with decreased QT when comparing days 3 and seven was 80%.

In addition, Kemp et al. [27] evaluated RFCSA in patients undergoing aortic surgery and demonstrated losses greater than 10% by day 7, documenting alterations in metabolome and proteome pathways. In this study, the reduction in RFCSA was greater, with the most significant difference observed between days 1 and 7.

Finally, Wandrag et al. [28] studied the relationship between catabolic status and loss of muscle mass evaluated by ultrasound in different muscle groups in patients with prolonged critical illness, finding an approximate 1.2% loss of muscle depth per day during a 14-day follow-up. Shen et al. [29], demonstrated in patients undergoing coronary revascularization and evaluated through tomography that postoperative loss of less than 5% in the skeletal muscle index was associated with worse survival. This finding suggests that preserving skeletal muscle mass is of paramount importance for patient recovery and prognosis following such surgical procedures. Tomography was utilized to assess and quantify skeletal muscle mass and its postoperative changes, offering valuable insights into the relationship between muscle mass and survival outcomes in these patients.

The current study conducted a multiparametric evaluation of measures related to quantitative and qualitative characteristics of skeletal muscle, including the sarcopenia index as a novel measure for sarcopenia analysis in elderly patients, and the hip score of muscle echogenicity, yet only significant reduction was demonstrated in one quantitative (RFCSA) and one qualitative (RFPA) variable.

Despite a standardized early rehabilitation process, a short duration of ventilatory and vasopressor support, the changes in the variables measured by ultrasound are greater compared to previous publications. Given the heterogeneity in results published by different groups, it is questioned whether, as in other types of critical illnesses, there may be treatable subphenotypes, endotypes, or treatable traits involved in skeletal muscle impairment in critically ill patients.

As limitations of this study, we acknowledge the small sample size, which may have contributed to the lack of statistically significant differences between the follow-up days for the evaluated variables; however, the post hoc analysis of the sample size justifies the analysis performed. A potential limitation is the ultrasound evaluation in a specific population, and the fact that ultrasound follow-up was limited to the 7th postoperative day. In addition, it was conducted in a single center so the results should be validated in multicenter studies with different populations.

Assessment of frailty is complex, and a multimodal approach is likely to be required. However, it is important to note that the present investigation adds evidence to the growing field of bedside ultrasound. We used multiple ultrasound measurements, considering that muscle involvement is a complex and heterogeneous process and that a single measurement may not be sufficient. We evaluated a specific group of patients with cardiac pathology in whom loss of muscle mass and function is common [30]. The approach of early identification of patients who lose muscle mass with greater speed or magnitude to establish early and precise actions is valid.

Therefore, we recommend promoting future research aimed at determining whether the loss of muscle mass extends into the later phase and establishing a possible correlation between this loss and its impact on other muscle groups, such as the diaphragm, seeking connections with trajectories of frailty and physical well-being in the medium and long terms [31]. Similarly, it is important to delve into defining the ultrasound parameters that could affect prognosis and determine the role of ultrasound in monitoring therapeutic interventions.

## Conclusion

In patients undergoing cardiac surgery, muscle ultrasound proved to be a viable technique for monitoring skeletal muscle alterations during the early postoperative period. Significant changes in quantitative and qualitative skeletal muscle variables were observed, with a gradual loss of muscle mass during follow-up until postoperative day 7. These muscle changes were not associated with ICU stay or increased mortality at day 28.

## Data Availability

The data sets used and/or analyzed during the current study will be available from the corresponding author upon reasonable request.

## Abbreviations

BIA: Bioelectrical impedance analysis
RFPA: Rectus femoris pennation angle
VPLA: Vastus lateralis pennation angle
RFCSA: Rectus femoris cross-sectional area
CAM–ICU: Confusion assessment method for the intensive care unit
CV: Coefficient of variation
SD: Standard deviation
DEXA: Dual-energy X-ray absorptiometry
ESPEN: European society for clinical nutrition and metabolism
BMI: Body mass index
QT: Quadriceps thickness
GLIM: Global leadership initiative on malnutrition
RFVIT: Rectus femoris and vastus internus thickness
VLT: Vastus lateralis thickness
FL: Fascicle Length
VLFL: Vastus lateralis fascicle length
Max: Maximum
Med: Median
Min: Minimum
MRC: Medical research council
HS: Hekmat score
ICU: Intensive care unit
X̅: Mean
d_0_: Day 0
d_1_: Day 1
d_3_: Day 3
d_5_: Day 5
d_7_: Day 7
